# Particle Adsorption on Hydrogel Surfaces in Aqueous Media due to van der Waals Attraction

**DOI:** 10.1038/s41598-017-06257-1

**Published:** 2017-07-21

**Authors:** Naoko Sato, Yurina Aoyama, Junpei Yamanaka, Akiko Toyotama, Tohru Okuzono

**Affiliations:** Faculty of Pharmaceutical Sciences, Graduate School of Nagoya City University, 3-1 Tanabe, Mizuho, Nagoya, Aichi 467-8603 Japan

## Abstract

Particle adhesion onto hydrogels has recently attracted considerable attention because of the potential biomedical applications of the resultant materials. A variety of interactions have been taken advantage of for adsorption, including electrostatic forces, hydrophobic interactions and hydrogen bonding. In this study, we report significant adsorption of submicron-sized silica particles onto hydrogel surfaces in water, purely by van der Waals (vdW) attraction. The vdW forces enabled strong adhesions between dielectric materials in air. However, because the Hamaker constant decreases in water typically by a factor of approximately 1/100, it is not clear whether vdW attraction is the major driving force in aqueous settings. We investigated the adsorption of silica particles (diameter = 25–600 nm) on poly(acrylamide) and poly(dimethylacrylamide) gels using optical microscopy, under conditions where chemical and electrostatic adsorption is negligible. The quantity of adsorbed particles decreased on decreasing the Hamaker constant by varying the refractive indices of the particles and medium (ethyleneglycol/water), indicating that the adsorption is because of the vdW forces. The adsorption isotherm was discussed based on the adhesive contact model in consideration of the deformation of the gel surface. The present findings will advance the elucidation and development of adsorption in various types of soft materials.

## Introduction

Adhesion and removal of fine particles on surfaces^1, 2^ is an important issue in a variety of scientific and engineering fields, including semiconductor fabrication, surface coatings, food processing^3^ and biomedical sciences^4^. In the microelectronic industry, for example, particle removal is an essential process in the production of clean wafer surfaces^1^. These adhesions occur owing to various driving forces, including van der Waals (vdW) forces, electrostatic interaction, hydrogen bonding, hydrophobic interaction and covalent bonding.

Recently, adhesion of colloidal particles on soft polymers and gels has attracted considerable attention in the field of soft materials^5, 6^ and also for use in material and biomedical applications, such as artificial muscles^7^ and surgical uses^8^. Rose *et al*.^9^ reported that two polymer hydrogel sheets as well as two portions of viscera could be tightly attached by placing nanometer-scale silica particles between pieces of the material. Thus far various innovative gels have been developed in which particles adhere onto surfaces through different adhesion forces, e.g., Coulomb forces between oppositely charged gels and particles^10^.

On the other hand, vdW force is not typically regarded as a major driving force for particle adhesion on gels. This is partly because of the short range and weak nature of vdW forces, particularly in water, where the strength of the force is usually less than 1/100 of its strength in air. However, the magnitude of the vdW force relative to particle size increases significantly for micron- or submicron-sized materials, where it may produce sufficiently strong adhesion. For example, the ability of geckos to maintain contact with the vertical wall surfaces while climbing is attributed to strong vdW attractions between micron-scale setae on their fingers and the wall^11–13^. Furthermore, the stability of aqueous colloids is generally determined by the balance between the magnitudes of repulsive forces and vdW attractions between the particles^14^.

**Figure 1:**
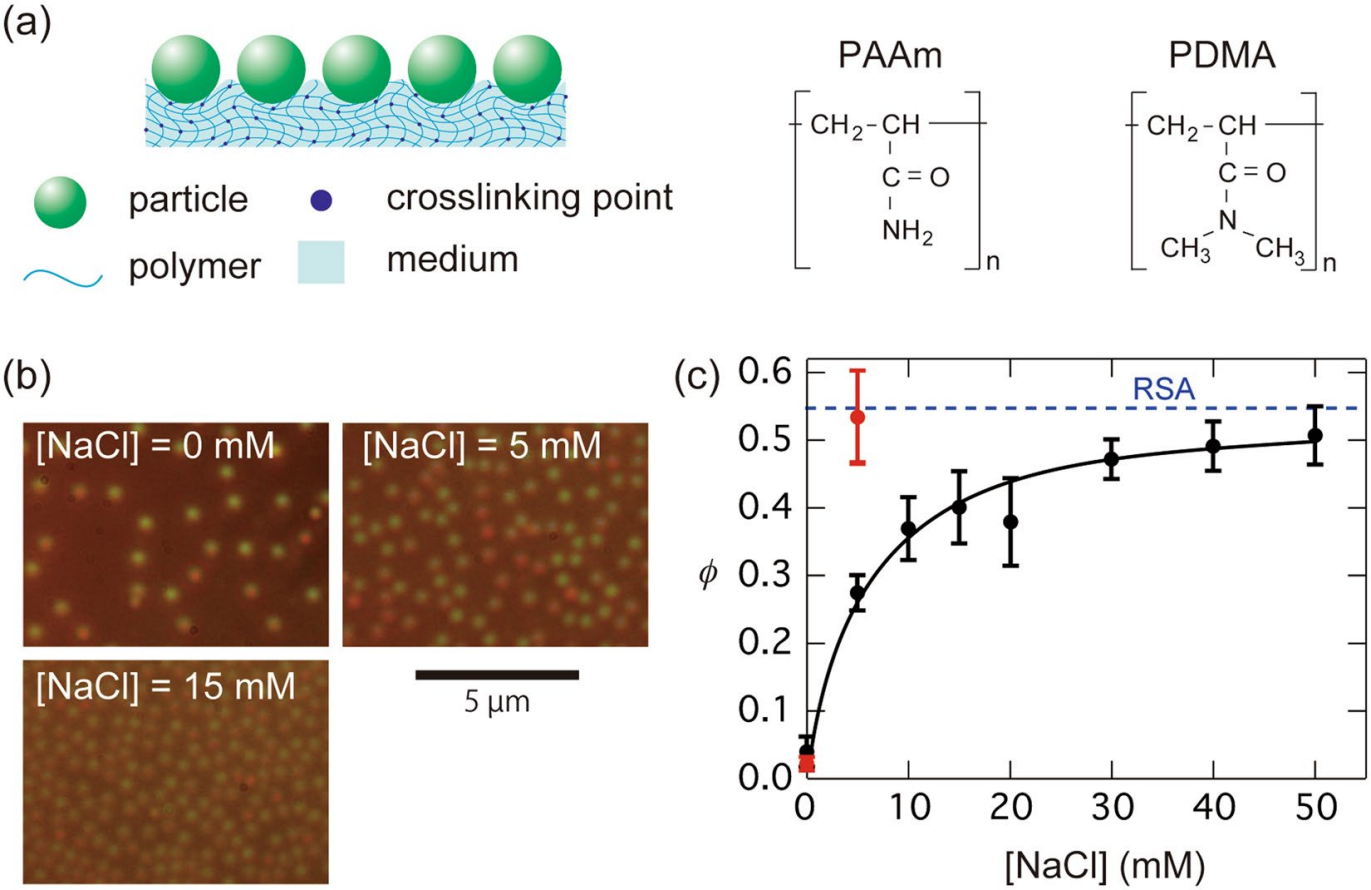
(**a**) An illustration of particle adsorption on polymer hydrogel surfaces, and the chemical structures of the two kinds of gel used. (**b**) Optical micrographs showing adsorbed colloidal silica particles (silica 1) on polyacrylamide (PAAm) gels in water at three different NaCl concentrations, [NaCl]. (**c**) Area fraction *ϕ* of the adsorbed silica particles in water plotted as a function of [NaCl] for PAAm and PDMA gels (black and red circles, respectively). Error bars represent standard deviations. The dashed horizontal line represents the maximum coverage (=0.547) for random sequential adsorption (RSA).

In this study, we report that colloidal particles can strongly adhere to hydrogel surfaces in water, because of vdW attractions (The adhesion of materials onto a liquid/solid interface is typically referred to as adsorption; here we use the term “adsorption” to indicate the adhesion of particles onto a gel surface in a liquid). Figure 1(a) illustrates the adsorption between particles and polymer hydrogel surfaces. Colloidal silica particles were used as they are widely used materials in colloid sciences and because various previous studies have experimentally investigated their adsorption capacities onto gels^9, 10^. Two types of vinyl polymer hydrogels, poly(acrylamide) [PAAm] and poly(dimethylacrylamide) [PDMA], were used and the influence of electrostatic interactions, Hamaker constant, and particle size on the adsorption behavior was determined. The observed adsorption behavior was explainable in terms of the vdW force, taking the deformation of the gel surfaces into accounts.

## Results and Discussion

### Adsorption of silica particles on gels: influence of particle charges

In this subsection, we report the adsorption of colloidal silica particles onto gel surfaces in pure water. The properties of the particles (silica 1) and the experimental system (system A) are shown in Tables 1 and 2, respectively. In Table 1, *d* is the particle diameter determined by the dynamic light-scattering method. *σ* and *Z* are charge densities and charge numbers of the particles. Details on the method used to characterize colloidal particles are described in the Materials and Methods section.

**Table 1:** Characteristics of the silica particles used in the study.

Sample	*d* (nm)	*−*σ (µC/cm²)	*−Z*
silica 1	550	0.11	6500
silica 2*	510	0.22	10300
silica 3*	108	0.16	320
silica 4*	71	0.22	210
silica 5*	25	0.13	16

*Labelled with green fluorescent dye.

**Table 2:** Composition of the experimental systems used in the study.

System	particle	*C*_p_ (vol%)	gel	[Bis] (mM)	medium	[NaCl] (mM)
A	silica 1	0.1	PAAm	10	water	0~50
B	silica 2	0.01	PAAm, PDMA	10	EG + water	50
C	silica 1	0.1	PAAm	2.5~40	water	50
D	silica 2	0.1	(quartz)	—	water	50
E	silica 3, 4, 5	0.01	PAAm	10	EG + water	50

1.0-mm-thick PAAm hydrogel sheets were synthesized from AAm (monomer) and *N*,*N*′-methylenebis-acrylamide (Bis, cross-linker) through photoinduced radical polymerization and cut into disk-shaped gels of 10 mm diameter. These were immersed in an aqueous dispersion of silica particles of particle concentration *C*_p_ = 0.1 vol% at different sodium chloride (NaCl) concentrations, [NaCl]. The samples were shaken for 2 h using an automatic shaker in a room maintained at 25 °C. The gel surfaces were washed in water to remove the non-adsorbed particles and were then observed in an inverted optical microscope. The sample preparation and adsorption experiment procedures are described further in the Materials and Methods section.

The addition of NaCl significantly increased the quantity of adsorbed particles. The micrographs shown in Fig. 1(b) demonstrate the influence of [NaCl], on the adsorption behaviour. From the micrographs, we calculated the area fraction *ϕ* of the adsorbed particles as *ϕ* = *ρ*_A_ *s*_p_, where *ρ*_A_ is the area number density of adsorbed particles and *s*_p_ = *πa*_p_^2^ (*a*_p_ is the particle radius) is the cross-sectional area of each particle. The *ϕ* values thus determined were plotted against [NaCl] (Fig. 1(c), black circles). The *ϕ* values increased with increasing [NaCl] and gradually plateaued at an [NaCl] value of approximately 30–50 mM. Because PAAm gels are not charged, the observed effect on [NaCl] appears to be caused by the electrostatic screening by NaCl for Coulomb repulsions between the non-adsorbed particles in the medium and adsorbed particles on the gel surfaces.

We note that PAAm gels sometimes bear slight negative charges because of the hydrolysis of acrylamide into acrylate anion (-CONH_2_ + H_2_O → -COO^−^ + NH_4_^+^). However, in separate experiments performed to determine the adsorption of positively charged polystyrene particles (*d* = 420 nm, *Z* = 5083), we observed that the *ϕ* value of the adsorbed particles did not differ remarkably from that of the silica particles (*ϕ* = 0.05 under salt-free condition). Thus, the influence of particle charge on the gels used herein, if any, was negligible. We investigated the influence of particle charge on the gels using poly(acrylamide-acrylic acid) copolymer gels, which is described in Supplementary Information A.

The *ϕ* value at the plateau in Fig. 1(c) was approximately 0.50, which is much smaller than the *ϕ* values for the two-dimensional (2D) closest-packing (*ϕ* = 0.91)^15^ and 2D random close-packing (0.81) of equal-sized spheres^16^. The smaller observed *ϕ* values may be partly attributed to the weak electrostatic repulsion between the silica particles, which afforded short gaps even at [NaCl] = 50 mM. On the other hand, Adamczyk *et al*.^17, 18^ reported that the irreversible adsorption (deposition) of particles onto planes could be regarded as random sequential adsorption (RSA)^19, 20^. The maximum coverage for RSA is 0.547 (represented in Fig. 1(c) by a dashed horizontal line)^19, 20^, which is close to the present plateau value. This suggests that the particle deposition on the gel surface was caused by a strong interaction other than Coulomb force.

The microscopic observation revealed that 94.5% of the silica 1 particles used for the adsorption experiments with [NaCl] = 50 mM were isolated and free ﻿in dispersions, whereas only 3.9% and 1.6% were present as dimers and aggregates of more than three particles, respectively. At [NaCl] values higher than 50 mM, the silica particles formed large aggregates in the dispersions (because of the vdW forces acting between the particles), which prevented us from obtaining the exact estimates of the adsorption quantities. Therefore, we used colloid samples with [NaCl] = 50 mM in all of the following experiments with gels.

We also examined the adsorption of silica 1 particles onto PDMA gels at two [NaCl] values [shown by the red symbols in Fig. 1(c)]. The *ϕ* value of PDMA was larger than that of PAAm and was close to the maximum coverage expected from RSA at [NaCl] = 5 mM. The observed stronger adsorption for PDMA gels appears to be attributed to hydrogen bonding^9, 21^ between the PDMA gel and silica in water along with the larger Hamaker constant of PDMA compared to PAAm. We further discuss the difference in adsorption behaviour between the two gels in the following sections.

### Tuning of van der Waals attraction between silica particles and gels

Next, we examined the adsorption mechanism between silica and hydrogels in greater detail. Interactions other than vdW forces, including hydrogen bonding and hydrophobic interactions, can also act as driving forces for adsorption. However, hydrogen bonding between silica and PAAm has only been reported to occur when the silica surfaces were insufficiently hydrated^22^. Adsorption by hydrogen bonding can be reasonably ruled out for our experiments because the silica used was fully hydrated by extending its contact with water.

In addition, because both PAAm and silica surfaces are highly hydrophilic, hydrophobic interactions have a negligible contribution. We performed experiments using a PAAm linear polymer (molecular weight = 4.3 × 10^5^) for adsorption onto silica surfaces in water using centrifuge and filtration methods. The adsorption experiments are described in detail in the Materials and Methods section. The resulting observed adsorption quantity was close to zero (1 ± 1%(s.d.)), whereas the particle surface area in total was 50–100% of that calculated for fully coverage by the PAAm chains (the radius of gyration = 40 nm). This indicates that the chemical adsorption between PAAm and silica is negligible.

Hence, the interpretation that the particle adsorption onto the gels was caused by vdW attractions is plausible. The vdW force include forces (i) between two permanent dipoles (Keesom force), (ii) between a permanent dipole and a corresponding induced dipole (Debye force), and (iii) between two instantaneously induced dipoles (London dispersion force)^14, 15, 23^. For a spherical particle near a flat surface, the interaction potential is given by:

1 $${U}_{{\rm{vdW}}}(x)=-\frac{A}{6}[\frac{{a}_{p}}{x}+\frac{{a}_{p}}{2{a}_{p}-x}+\,\mathrm{log}(\frac{x}{2{a}_{p}-x})],$$

where *x* is the distance of the sphere from the surface. *A* is the Hamaker constant, which is related to the refractive index *n* and the relative electrical permittivity *ε* of the materials in the system. At sufficiently small separation,

2 $${U}_{{\rm{v}}{\rm{d}}{\rm{W}}}(x)=-\frac{A}{6}\frac{{a}_{p}}{x}$$

According to the Lifshitz theory, the Hamaker constant can be estimated by accounting for the frequency (*v*)-dependence of the permittivity^15^. For the two dielectric materials 1 and 2 interacting across a medium *m*, *A* is the sum of the zero-frequency term (*A*_*v*=0_), due to the Keesom and Debye forces, and nonzero-frequency term (*A*_*v*>0_) resulted from the London force by a thermal fluctuation of frequency *v*. They are respectively expressed as

3 $${A}_{\nu =0}=\frac{3{k}_{{\rm{B}}}T}{4}\frac{{\varepsilon }_{1}-{\varepsilon }_{m}}{{\varepsilon }_{1}+{\varepsilon }_{m}}\frac{{\varepsilon }_{2}-{\varepsilon }_{m}}{{\varepsilon }_{2}+{\varepsilon }_{m}}$$

and

4 $${A}_{\nu  > 0}=\frac{3h{\nu }_{e}}{8\sqrt{2}}\frac{({n}_{1}^{2}-{n}_{m}^{2})({n}_{2}^{2}-{n}_{m}^{2})}{{({n}_{1}^{2}+{n}_{m}^{2})}^{\frac{1}{2}}{({n}_{2}^{2}+{n}_{m}^{2})}^{\frac{1}{2}}[{({n}_{1}^{2}+{n}_{m}^{2})}^{\frac{1}{2}}+{({n}_{2}^{2}+{n}_{m}^{2})}^{\frac{1}{2}}]},$$

where *k*_B_ and *T* are the Boltzmann constant and temperature, respectively, and *h* is the Planck constant. *v*_e_ is the main electronic adsorption frequency, for which we used a typical value *v*_e_ = 3.0 × 10^15^/s [ref. 15].

It should be noted that London dispersion force is reduced on increasing separation, due to the retardation effect. Gregory^24^ reported an approximate expression for the retarded dispersion force, which gives *A*_*v*>0_ (*x*) = *A*_*v*>0_ (0)/(1 + 14*x*/*λ*), where *λ* = *v*_e_/*c* (*c* is the velocity of light). The approximated interaction energy closely agree with an exact calculation based on Lifshitz theory for *x* < 100 nm. The Keesom and Debye interactions remain non-retarded at all separations. However, in the presence of electrolytes, they are reduced because of electric conduction effect^14, 15^. *A*_*v*=0_(*x*) is then given by

5 $${A}_{v=0}(x)={A}_{v=0}(0)\exp (-\kappa x),$$

where *κ* is the Debye parameter defined as *κ* ^2^ = (*e*_0_/*ε*_r_ *ε*_0_)*I*, where *e*_0_ is the elementary charge and *I* = ∑*z*_*j*_^2^ *c*_*j*_ is the ionic strength of the medium (*z*_*j*_ and *c*_*j*_ are the valences and concentrations, respectively, of the *j*-th small ion). In the following analysis we will use a short-range expression of the retarded vdW potential taking the salt effect into accounts, i.e.,

6 $${U}_{{\rm{vdW}}}(x)=-[{A}_{\nu =0}(0)\exp (-\kappa x)+{A}_{\nu  > 0}(0)/(1+\frac{14x}{\lambda })]\frac{{a}_{p}}{6x}$$

Hereafter, the values of *n* and *ε* for silica and gel are denoted by the subscripts 1 and 2, respectively. *ε*_2_ was estimated as the volume average of the values for PAAm and the medium^25, 26^.

7 $${\varepsilon }_{2}=(1-{\varphi }_{polym}){\varepsilon }_{m}+{\varphi }_{polym}{\varepsilon }_{polym},$$

where *ϕ*_*polym*_ (=0.084) is the volume fraction of polymer in the gel and *ε*_*polym*_ is the permittivity of PAAm (=5.6)^27^. It should be noted that *A*_*v*=0_ and *A*_*v*>0_ are proportional to the differences between the *ε* and *v* values of the materials (particle or gel) and the medium. Therefore, the vdW forces will be weaker when the refractive index and permittivity of the materials are closer to those of the medium.

**Figure 2:**
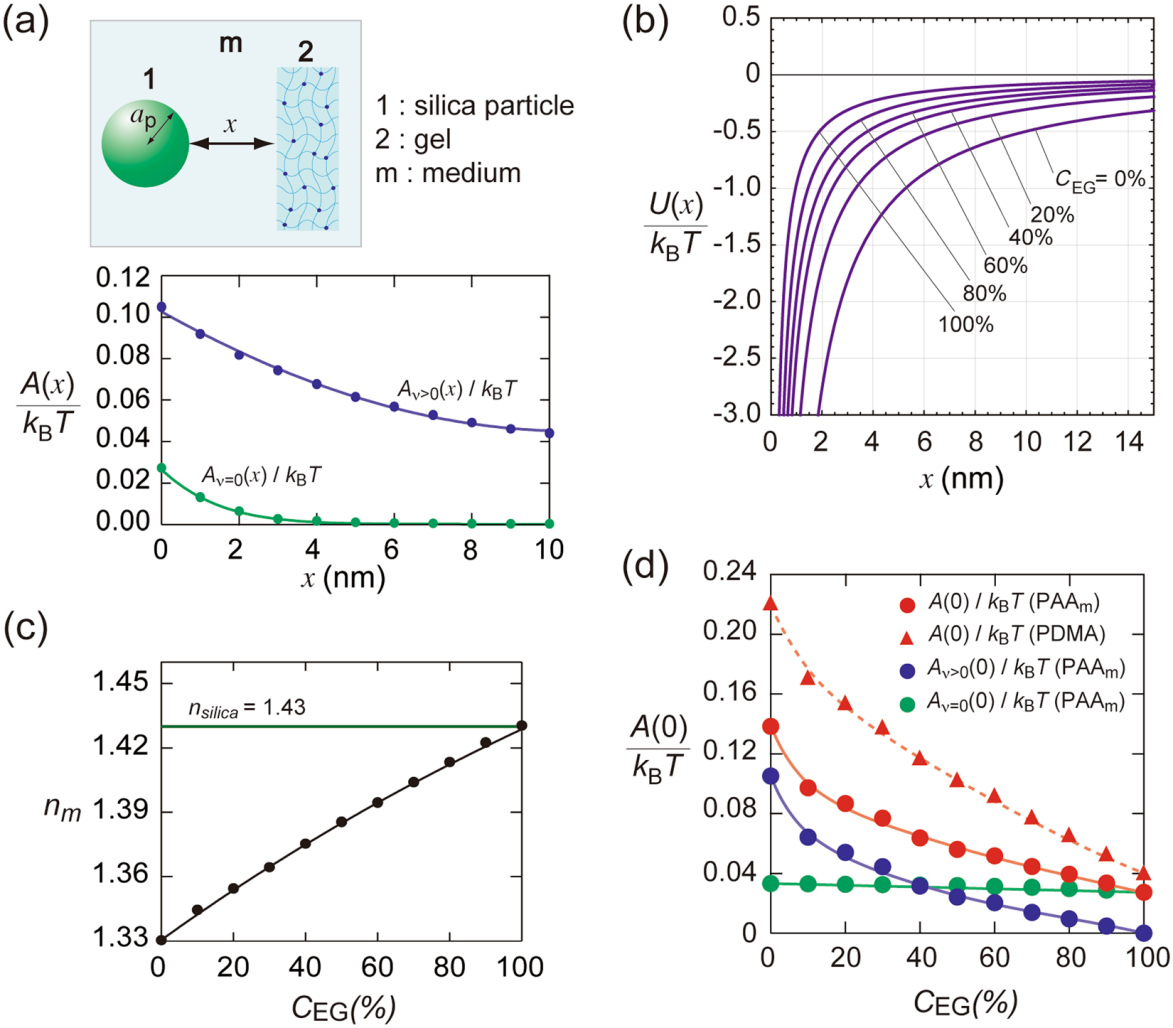
(**a**) Zero-frequency (${A}_{\nu =0}$), and nonzero-frequency (${A}_{\nu  > 0}$) terms of the Hamaker constant as functions of separation *x*; silica1 particles and PAAm gel system in water with [NaCl] = 50 mM. (**b**) Calculated vdW potential between silica 1 particles and PAAm gel at various values of ethylene glycol (EG) concentrations, *C*_EG_. (**c**) Refractivity of aqueous solutions of EG at different values of *C*_EG_. (**d**) Plot of the Hamaker constant *vs. C*_EG_ estimated from Lifsitz theory (eqs 3 and 4).

For the given values of *n*_1_ (=1.43, according to the manufacturer) and *ε*_1_ (=3.8), and *n*_*m*_ = 1.33 and *ε*_*m*_ = 78.3 for water at 25 °C, we obtain a vdW attraction of *A*(0) = 0.14 *k*_B_ *T* [*A*_*v*=0_ (0) = 0.03 *k*_B_ *T* and *A*_*v*>0_ (0) = 0.11 *k*_B_ *T*] between the silica particles and gel in water. This value is remarkably smaller than that in air (*n*_*m*_ = *ε*_*m*_ = 1), where *A* (0) = 11.3 *k*_B_ *T* [*A*_*v*=0_ (0) = 0.43 *k*_B_ *T* and *A*_*v*>0_ (0) = 10.8 *k*_B_ *T*], because the values of (*ε*_1_ − *ε*_*m*_) (*ε*_2_ − *ε*_*m*_) and (*n*_1_ − *n*_*m*_) (*n*_2_ − *n*_*m*_) are much smaller in water. Figure 2(a) shows variations of *A*_*v*=0_ and *A*_*v*>0_ with *x*. Here *A*_*v*=0_ was calculated for aqueous solution of 50 mM NaCl at 25 °C (1/*κ* = 1.38 nm). *A*_*v*=0_ value decays rapidly on increasing *x* and is less than 0.001 *k*_B_ *T* for *x* > 5 nm. The vdW potential curve in water is shown in Fig. 2(b), indicating that the vdW attraction is sufficiently strong at a small distance from the PAAm gel surfaces.

The Hamaker constant is tunable by varying the refractive index of the medium. Ethylene glycol (EG) has a value of *n*_*m*_ = 1.43 at 25 °C, which is nearly the same as that of the silica used here. Figure 2(c) shows the values of *n*_*m*_ for aqueous solutions of EG at various concentrations, *C*_EG_, taken from literature^28^. The equilibrium volume of the PAAm gel does not vary significantly with changes in *C*_EG_^29^ (see also Supplementary Information B), which notably facilitates the data analysis. The values of Hamaker constant estimated from eqs (3) and (4) for different values of *C*_EG_ are shown in Fig. 2(d). The *ε*_*m*_ values were taken from the literature^30^, and the *n*_2_ values were measured using an Abbe refractometer for different *C*_EG_ values at 25 °C (*n*_2_ = 1.35 for 1.33 M aqueous solution). The value of *A*(0) (=*A*_*v*=0_(0) + *A*_*v*>0_(0)) thus calculated is shown in Fig. 2(d). *A* (0) significantly decreased with increasing *C*_EG_ owing to the large decrease in *A*_*v*>0_(0), but did not reach zero for any value of *C*_EG_ because of the contribution of *A*_*v*=0_(0). *A*_*v*=0_(*x*) decays with increasing *x*, and *A*_*v*=0_ ≈ 0 at *x* = approximately 5 nm. Thus for *x* > 5 nm and at high *C*_EG_, *A* ≈ *A*_*v*=0_ is approximately zero. The potential curves at various values of *C*_EG_ are also shown in Fig. 2(b). The vdW attraction decreased remarkably and became short-ranged with increasing *C*_EG_.

### Adsorption of silica particles on gels due to van der Waals attraction

**Figure 3:**
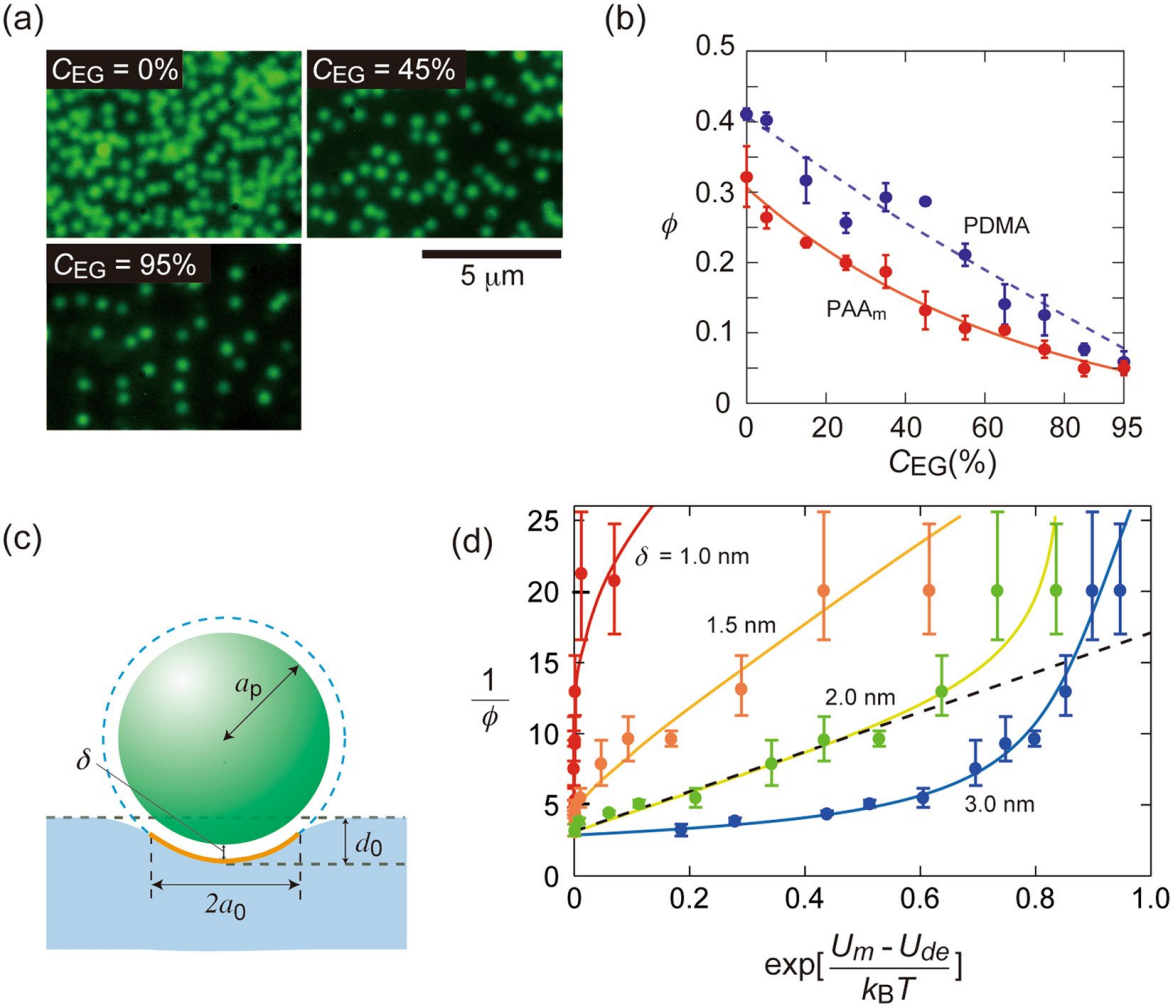
(**a**) Micrographs of fluorescent silica 2 particles adsorbed on PAAm gels at three values of *C*_EG_, with [NaCl] = 50 mM. (**b**) *ϕ* vs *C*_EG_ plots for particle adsorption onto PAAm and PDMA gels. (**c**) An illustration of deformation of gel on adhesive contact of a rigid particle. (**d**) 1/*ϕ vs*. exp[(*U*_m_ − *U*_de_)/*k*_B_ *T*] plot at various *C*_EG_s. Error bars in panels (**b** and **d**) represent standard deviations.

We examined the adsorption of silica particles onto PAAm gel at various *C*_EG_ values (system B in Table 2). Here, we used fluorescent-labelled silica 2 particles to obtain micrographs of individual particles even under nearly index-matched conditions. Figure 3(a) displays micrographs of the adsorbed particles at three *C*_EG_ values. The *ϕ* value for the adsorbed particles is plotted against *C*_EG_, represented using red symbols in Fig. 3(b). The *ϕ* values notably decreased with increasing *C*_EG_, which strongly suggests that the particle adsorption was mainly governed by vdW forces.

We further study the adsorption of silica particles onto PDMA gels (*ϕ*_*polym*_ = 0.134). The size of PDMA gel for different values of *C*_EG_ was nearly constant (Supplementary Information B). The Abbe refractometer was used to measure the *n* values of the PDMA gels, which exhibited somewhat larger *n* values than PAAm (*n*_2_ = 1.36 for 1.33 M aqueous solution). *ε* of PDMA from literature was 3.6^31^. The Hamaker constant between silica and PDMA across the medium is approximately 20% larger than that of PAAm in water. The *A* value for PDMA gel is shown in Fig. 2(d) using red triangles. The observed *ϕ* for PDMA is shown in Fig. 3(b), which is larger by more than 20% than that for PAAm. We note that several authors have assumed that the attraction between PDMA and silica is stronger than that between PAAm and silica because of the hydrogen bonds formed in the case of PDMA^9, 21^. In our opinion, the greater adsorption onto PDMA can also be explained by a stronger vdW attraction, because for sufficiently high *C*_EG_ values, where PAAm and PDMA have similar *A* values, the quantity of particles adsorbed onto each gel is similar.

We estimated *ϕ* of the adsorbed particles from the vdW potential as follows, taking elastic deformation of the gel surfaces into accounts. The Hertz contact theory^32^ provides a pressure and shapes for the elastic deformation of two bodies in contact without adhesion. Johnson–Kandall–Roberts (JKR)^33^ and Derjaguin–Muller–Toporov (DMT)^34^ have reported theories on the adhesion of two elastic bodies in contact. DMT theory assumed that the contact profile remains the same as in Hertzian contact but with additional attractive interactions. Later it was shown by Tabor^35^ that these two theories were the extreme limits of a single theory; that is, the JKR theory applies to soft materials with large surface energies and radii, whereas the DMT theory is valid for hard solids with small radii and low surface energies. Maugis^36^ reported a general theory for contact adhesion between an elastic sphere and an elastic half-space (hereafter denoted as 1 and 2, respectively) based on the Dugdale model. A continuous transition between the JKR and DMT theories was found on varying a parameter *λ* defined as

8 $$\lambda =2{\sigma }_{0}{[{a}_{{\rm{p}}}/(\pi {K}^{2}W)]}^{1/3}$$

Here, *σ*_0_ is the magnitude of Dugdale stress; *K* = (4/3)[(1 − v_1_^2^)/*E*_1_ + (1 − v_2_^2^)/*E*_2_]^−1^, where *E*_1_ and *E*_2_ are the Young’s moduli of 1 and 2, respectively, and v_1_ and v_2_ are the Poisson’s ratios of 1 and 2, respectively; and *W* is the interfacial free energy per unit area. The Maugis–Dugdale (MD) model becomes equivalent to the DMT and JKR models at the limits of *λ* = 0 and *λ* = ∞. Generally, the DMT and JKR theories are regarded to be valid for *λ* < 0.1 and *λ*  > 5, respectively^35, 37^.

For the silica sphere + flat gel system, we can assume *E*_1_ ≫ *E*_2_. Because our gels were composed of nearly all liquid (≈90%) and were nearly incompressible, v_2_ ≈ 1/2. That is, *K* ≈ (4/3)^2^ *E*_2_. From stress–strain measurements, we obtained *E*_2_ = 6.3 kPa for the PAAm gel used in the experiments shown in Fig. 3(d). By using the short-range expression of vdW interaction, we have *W* = *A*/(12π*δ* ^2^) (ref. 24), where *δ* is the minimum separation distance between the sphere and plane. Thus, for a given value of *δ*, we can evaluate *λ* at various values of *C*_EG_ based on *A*(*δ*) at a given *C*_EG_. The *λ* versus *C*_EG_ curves are presented in Fig. S5 of Supplementary Information C.

According to the JKR theory, the contact radius in the absence of an external force, *a*_0_, is equal to [6π*Wa*_p_^2^/*K*]^1/3^, whereas the DMT theory gives *a*_0_ = [2π*Wa*_p_^2^/*K*]^1/3^ (ref. 36). In the MD theory, *a*_0_(*λ*) is given in the form of *a*_0_(*λ*) = α(*λ*)[π*Wa*_p_^2^/*K*]^1/3^, where α(*λ*) is a parameter that increases from α(0) = 2^1/3^ to α(∞) = 6^1/3^. Carpick *et al*.^38^ derived the following approximate formula for α(*λ*): α(*λ*) = 1.54 + 0.279(2.28*λ* ^1.3^ − 1)/(2.28*λ* ^1.3^ + 1). An indentation of the sphere into the gel, *d*_0_ [Fig. 3(c)], is obtained as *d*_0_ = *a*_p_ [1 − √(1 − (*a*_0_/*a*_p_)^2^)]. The *a*_0_ and *d*_0_ versus *C*_EG_ curves at three values of *δ* are shown in Fig. S5 of Supplementary Information C. For *δ* = 1 nm and low *C*_EG_ (*λ* > 5), the values obtained by the MD theory were close to those obtained by the JKR theory. In contrast, at *δ* = 3 nm and high *C*_EG_ (*λ* < 0.1), the DMT theory provided a good approximation of the MD theory.

Recently, Style *et al*.^6^ studied adhesion contact between silica particles and soft surfaces (silicone plates) in air. They reported that the JKR theory well described the adhesion contact with soft surfaces when the effect of surface tension was accounted for. Because the gel surfaces in our system were immersed in the same liquid as the gel medium, the interfacial energy between them was negligibly small. In that case, the total energy of adhesive contact is the sum of the Hertz elastic energy *U*_*el*_ and adhesion energy between the particle and gel *U*_*a*_. Using the JKR formalism, we obtained *U*_*el*_ = 8/(5√3)*E*_2_ *a*_p_^1/2^ *d*_0_^5/2^/(1 − *v*_2_^2^) and *U*_a_ = −π*a*_p_^2^ *W*. We assumed the energy required for the desorption of a particle, *U*_*de*_, to be given by *U*_*de*_ = *U*_*el*_ + *U*_*a*_. Figure S7 of Supplementary Information C presents the values of *U*_*el*_, *U*_*a*_, and *U*_*de*_ at various values of *C*_EG_.

The values of *δ* determined by measurements of surface force usually lie in the range of 1 to 3.5 nm because of solvation effects^15^. As an example, if we assume *δ* = 1 nm, we obtain *a*_0_ = 106 nm (*d*_0_ = 15 nm) and *E* = 71 *k*_B_ *T* for the adsorption of silica 1 onto PAAm gel in water. This value is much larger than that for the vdW energy (without gel deformation) at *δ* = 1 nm (=4.4 *k*_B_ *T*), suggesting that the desorption of the particles from the soft gel surface requires a much larger energy than their desorption from a hard surface.

Based on the adhesion contact theory mentioned above, we derived a Langmuir-type adsorption isotherm of particles onto gel surfaces. The rates of particle adsorption, *R*_*ad*_, is determined by the collision frequency of particles with free surface, according to *R*_*ad*_ = *k*_1_ *ρ* (ϕ* − *ϕ*). Here *k*_1_ is a constant, *ρ* the number concentration of particles, and ϕ* the area fraction of particles at a maximum adsorption. On the other hand, the desorption rate, *R*_*de*_, is represented as *R*_*de*_ = *k*_2_ *ϕ* exp(−*U*_*de*_/*k*_B_ *T*), by assuming Arrhenius type desorption. Here *U*_*de*_ is an activation energy, and *k*_2_ a constant. Equating *R*_*ad*_ and *R*_*de*_ yields

9 $$\varphi =\frac{\rho {\varphi }^{\ast }}{\rho +K^{\prime} \exp (-{U}_{de}/{k}_{{\rm{B}}}T)}$$

where *K*′ = *k*_2_/*k*_1_. When the vdW interaction is at work, *ρ* (*x* = *δ*) is provided by assuming Boltzmann distribution *ρ* = *ρ*_0_exp(−*U*_*m*_/*k*_B_ *T*), where *U*_*m*_ = *U*(*δ*) is the vdW energy at the closest approach (potential minimum), and *ρ*_0_ is the *ρ* value in bulk. Thus,

10 $$\varphi =\frac{{\rho }_{0}{\varphi }^{\ast }}{{\rho }_{0}+K^{\prime} \exp [({U}_{m}-{U}_{de})/{k}_{{\rm{B}}}T]}$$

or,

11 $$\frac{1}{\varphi }=\frac{1}{{\varphi }^{\ast }}+(\frac{K^{\prime} }{{\rho }_{0}{\varphi }^{\ast }})\,\exp [\frac{{U}_{m}-{U}_{de}}{{k}_{{\rm{B}}}T}]$$

In Fig. 3(d), 1/*ϕ* values observed at various *C*_EG_ are plotted against exp[(*U*_m_ − *U*_de_)/*k*_B_ *T*] at four values of *δ* (Curves in Fig. 3(d) are guide for eyes). The error bars in Fig. 3(d) are estimated from the standard deviation of the observed values of *ϕ*. A good linearity was observed for *δ*  = 2 nm, except two data points of large experimental errors obtained at high *C*_EG_ (shown by a dashed line in Fig. 3(d)). Thus, the particle adsorption onto the PAAm hydrogels was explainable in terms of Langmuir-type adsorption taking the deformability of gel surfaces into accounts.

**Figure 4:**
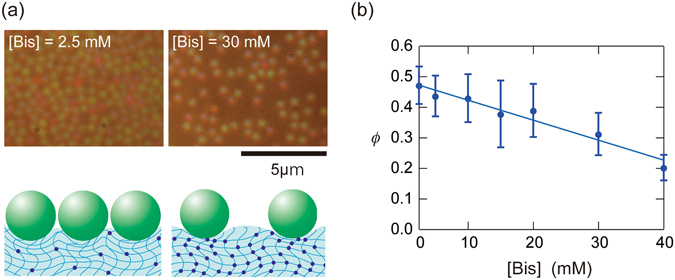
(**a**) Micrographs of adsorbed silica 1 particles on PAAm gel prepared for two values of [Bis] in water, and illustration of the adsorption states; (**b**) *ϕ* vs. [Bis] plot. Error bars represent standard deviations.

Young’s modulus of the gel varies depending on the cross-linker (Bis) concentrations, [Bis], in the gel preparation. With increasing [Bis], the average distance between polymer chains in the gel decreases, making the gel more rigid^39^. Influence of [Bis] on the particle adsorption is shown in Fig. 4(a) and (b) (System C in Table 2). *K* values of the gel determined by rheology measurements were 4.1 kPa and 46 kPa, for [Bis] = 2.5 mM and 40 mM, respectively. Using higher [Bis] resulted in lower quantities of adsorbed particles, which appears to be attributable to a change in the deformability of the gel.

### Adsorption onto quartz plates

To verify the influence of vdW attraction on silica adsorption, we further examined the adhesion of the silica particles on solid quartz surfaces for different *C*_EG_ values. Quartz has a chemical composition identical to that of silica (SiO_2_) with a slightly larger refractive index (1.47) because of its higher density. The Hamaker constants, *A*(0), between silica and quartz across each medium (1.52 *k*_B_ *T* and 0.50 *k*_B_ *T*, in pure water and pure EG, respectively) are much larger than those for the gels.

**Figure 5:**
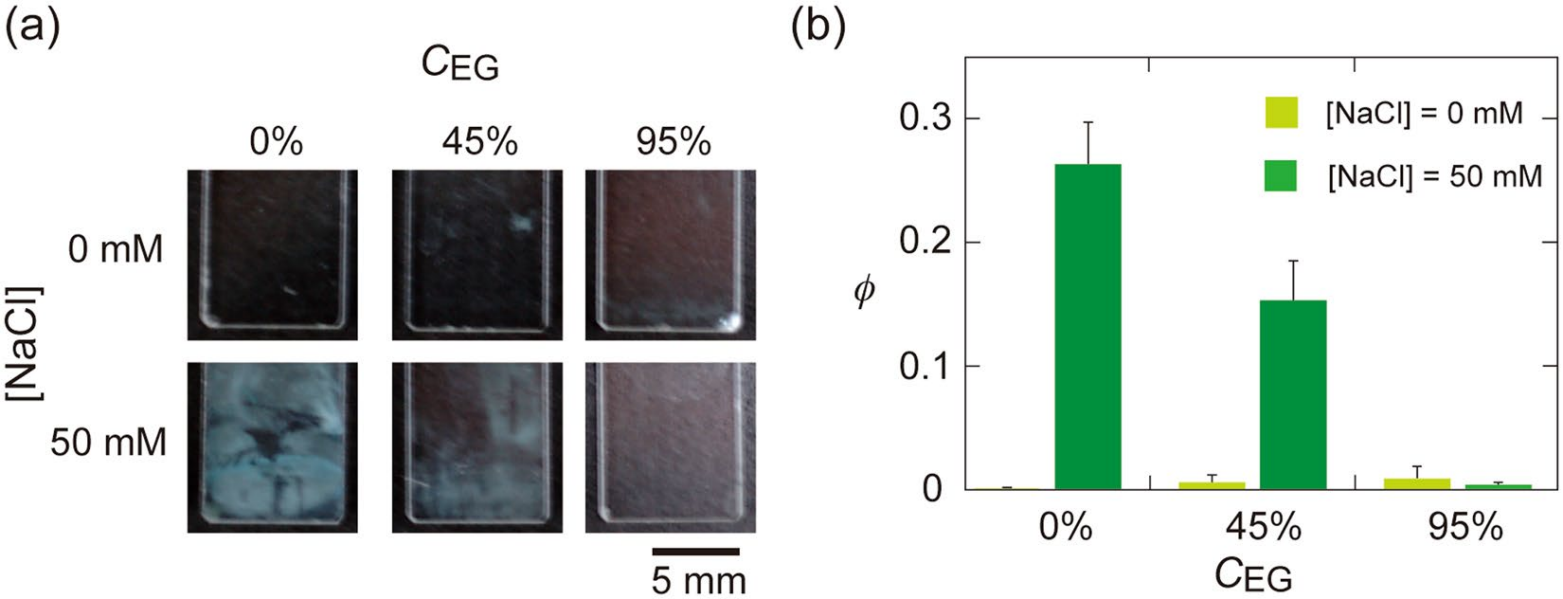
The influence of *C*_EG_ on the adsorption of silica 1 particles on quartz plates at two values of [NaCl]. Overviews of (**a**) the adsorption states and (**b**) *ϕ* values. Error bars represent standard deviations.

Figure 5(a) demonstrates the influence of *C*_EG_ on silica 1 adsorption onto quartz plates under salt-free conditions and at [NaCl] = 50 mM (system D). As seen for the silica–gel systems, adsorption was negligible under salt-free conditions because of the strong electrostatic repulsions between the silica and quartz plates. A strong reduction in adsorption with increasing *C*_EG_ was also observed for the quartz plate, indicating a significant contribution from vdW attractions between the particle and the gels in the aqueous systems.

### Influence of particle size

The *U*_vdw_(*x*) in eq. (1) approaches (−*A*/6) *a*_p_/*x* as *x* → 0 (eq. 2). That is, the vdW attractions between the adsorbed particles and gels decreases in proportion to the particle size. We examined the particle-size dependence of adsorption using fluorescent particles of *d* = 108 nm, 71 nm, and 25 nm (silica 3, 4 and 5; system E). Although single particles adsorbed onto the gel could not be detected by optical microscopy, we were able to estimate the extent of adsorption from the relative fluorescence intensity on the gel surfaces, which was 1:0.86:0.5. for silica 3, 4 and 5, respectively. Therefore, adsorption decreased for smaller particle sizes. This finding additionally supports the above-mentioned conclusion that vdW attraction is the major driving force for particle adsorption.

## Conclusions

We investigated the driving force for the adsorption of colloidal silica particles onto PAAm and PDMA hydrogels under salt-present conditions in which electrostatic interactions were minimized. Significant particle adsorption onto the gel surfaces was observed, although adsorption onto a linear polymer (PAAm) was negligible; that is, chemical adsorption between the particles and the gel can be safely disregarded. Thus, we conclude that the vdW attraction between the particles and gel was strong enough to enable adsorption in water, despite the much smaller Hamaker constant compared with the conditions in air. The results of the adsorption experiments in which the refractive index of the medium was varied suggest that the adsorption quantity decreases with decreasing values of the Hamaker constant. The adsorption behavior was explainable in terms of Langmuir-type adsorption isotherm taking the deformability of gel surfaces into accounts. In addition, smaller particles exhibited lower adsorbed quantities. Collectively, these findings strongly support the idea that vdW attraction is the primary driving force for particle adsorption. We expect the present findings to have important implications for the elucidation of adsorption properties of various gels and for the fabrication of novel gel materials.

## Materials and Methods

### Colloidal particles

The parameters of the colloidal samples used in this study are listed in Table 1. Silica 1 was purchased from Nippon Catalyst Co., Ltd., Osaka, whereas silica 2 to silica 5 samples were obtained from Polyscience Inc (Warrington, Pennsylvania). The silica 1 samples were dialyzed in cellulose tubes (pore size 2.4 nm) against purified water for 30 days, and then a mixed bed of cation- and anion-exchange resin beads (AG501-X8(D), Bio-Red Laboratories, Hercules, CA) was added to the sample, after which the samples were left to stand for one week for further ionization. The silica 2 to silica 5 samples were used for the adsorption experiments without further purification. The concentration of particles in each stock solution was determined by drying. Because of the silanol (Si–OH) groups on the particle surfaces and the dissolution of airborne carbon dioxide, the colloid samples were slightly acidic. At the particle concentration used in the adsorption experiments (0.1 vol%), the pH value of the samples was 5.48 ± 0.06. The water used had an electrical conductivity of 0.4–0.6 μS/cm after purification with a Milli-Q Integral system (Millipore, Massachusetts).

Particle diameters were determined using a dynamic light scattering (DLS) apparatus (FDLS-3000 system; Photal Co., Ltd., Osaka), equipped with a solid laser (100 mW, wavelength = 532 nm). The diffusion coefficients *D* of the particles were measured at particle concentration *C*_p_ ≤ 0.01% and at sodium chloride concentration [NaCl] = 10 μM. From *D*, the particle diameters *d* (Table 1) were estimated by the Stokes–Einstein equation. The *d* values obtained at [NaCl] = 100 μM for silica 1 and 2 were 541 ± 93 nm (s.d.) and 509 ± 114 nm (s.d.), respectively, which were not significantly different from those obtained at [NaCl] = 10 μM; the corresponding *d* values of these samples determined by optical microscopy were 565 ± 15 nm (s.d.) and 488 ± 14 nm (s.d.), respectively, in close agreement with the DLS results.

The aggregation of silica 1 particles in aqueous dispersions with [NaCl] = 5 mM was examined based on optical micrographs of more than 5,500 particles taken 2 h after sample preparation.

The electrophoretic mobility of the colloidal particles was measured by microscopic electrophoresis using a Zeecom system (Microtec Co., Ltd., Chiba, Japan). The particle number concentration was 10^9^–10^11^ L^−1^ and [NaCl] was 10 μM. The *ζ* values of silica 1 and silica 2 samples using the Henry equation from the averaged mobility for more than 100 particles were −15 mV and −29 mV, respectively. The effective surface charge densities *σ* and charge numbers *Z* of the particles were determined by electrical conductivity measurements using a previously reported method^40^. The conductivities of salt-free aqueous silica colloids were measured using a type DS-52 conductivity meter (Horiba Co., Ltd., Kyoto) and a conductivity cell with a cell constant of 1.00 cm^−1^. The samples were deionized by additions of the ion-exchange resin beads before use.

### Hydrogel synthesis

The hydrogels were prepared through photo-induced radical polymerization in an aqueous solution of gel monomers [acrylamide (AAm), or *N*,*N*′-dimethylacrylamide (DMA);1.33 M], a cross-linker *N*,*N*′-methylene bisacrylamide (Bis) and a polymerization initiator 2,2′-Azobis[2-methyl-*N*- (2-hydroxyethyl)propionamide] (VA-086, 0.35 mM). The concentration of Bis was varied as required. A total of 2 ml of the mixed solution was purged with Ar_2_ or N_2_ gas for 20 min.

### Particle Adsorption experiments

Disk-shaped gel samples were immersed in the colloidal dispersions and shaken for 2 h using an automatic shaker in a room thermostated at 25 °C. An inverted optical microscope (ECLIPSE 80i, Nikon) was used for the measurements. The area fraction of adsorbed particles was calculated by counting the number of particles adsorbed onto the gel, observed from 6–7 microscope images covering an area of at least 2240 μm^2^. We repeated all of the adsorption experiments three times, and the data are presented as average values with standard deviations (error bars).

### Experiments of PAAm linear polymer adsorption onto silica particles in water

PAAm linear polymers were purchased from Polysciences, Inc. The molecular weight was determined by viscosity measurements for a theta solvent (water:methanol = 3:2 vol/vol) at 20 °C using an Ubbelohde-type viscometer with a solvent efflux time of 236 s. The viscosity average molecular weight *M* was determined from the intrinsic viscosity [*η*] based on the Mark−Houwink−Sakurada equation [*η*] = *K*_v_ *M* ^*a*^, where *K*_v_ = 1.27 × 10^−4^ mg/mol and *a* = 0.5 for PAAm for the given conditions^41^.

An aqueous solution of PAAm and a silica 1 dispersion was mixed and given an ultra-sonication treatment for 5 min. The concentrations of PAAm and silica particles in the mixture were 0.1 wt% and 10 vol%, respectively. The total particle surface area was approximately 50%, as large as that expected for full coverage by the PAAm chains. A 5 mL sample was ultra-centrifuged in an Optima XE-90 centrifuge apparatus (Beckman Coulter, California) at 40,000 rpm for 60 min, to allow sedimentation of the silica particles. The temperature was controlled at 25 °C. The PAAm concentration in the supernatant was determined by UV adsorption at 230 nm by using a UV-VIS spectrophotometer (UV-2400PC; Shimadzu Co., Ltd., Kyoto). The experiments were performed at NaCl concentrations of 0, 0.01, 0.1, and 1 M.

We also determined the adsorbed quantity of PAAm by detecting the PAAm concentration in silica (diameter = 1.5 µm) + PAAm samples. The concentrations of the silica particles and PAAm were 10 vol% and 0.024 wt%, respectively. The total particle surface area was the same as that expected for full coverage by the PAAm chains. The samples were shaken for 2 h and filtered using hydrophilic poly(tetrafluoroethylene) membranes (pore size = 1 µm). The experiments were performed at [NaCl] = 0 and 1 M.

### Measurements of Young’s modulus of the gels

We used a RE-33005 rheometer (Yamaden Co., Ltd., Tokyo) to measure the stress–strain curves of the gels. The apparatus was equipped with a cylindrical bar (diameter = 8.00 mm). The strain values on applying given values of stress were measured.

## Electronic supplementary material

Supplementary Information
