# Supplementary material for: Particle Adsorption on Hydrogel Surfaces in Aqueous Media due to van der Waals Attraction
Source: Sci Rep. 2017 Jul 21;7:6099. doi: 10.1038/s41598-017-06257-1 (PMC5522479; doi:10.1038/s41598-017-06257-1)
Supplement: Supplementary file 1 — Supplementary Information [file 41598_2017_6257_MOESM1_ESM.pdf]

Supplementary Information

"Particle Adsorption on Hydrogel Surfaces in Aqueous Media due to van der Waals Attraction"

by Naoko SATO, Junpei YAMANAKA, Akiko TOYOTAMA, and Tohru OKUZONO

A. Interaction potential between charged particles and like-charged poly (acrylamide - acrylic acid) gel

We performed adsorption experiment of negatively charged polystyrene particles (a_p = 300 nm, Z = 3256) onto negatively charged poly (acrylamide - acrylic acid) [hereafter designated as P(AAm-AAc)] copolymer gel in water. P(AAm-AAc) gels were synthesized from acrylamide and acrylic acid monomers (molar ratio = 95 : 5).

The electrostatic interaction potential $U_e\ (x)$ between a charged sphere and a flat surface separated by a distance x is given by

$$U_e(x) = a_p \varepsilon_m \psi_1 \psi_2 \log[1 + \exp(-\kappa x)]$$

Here, ψ_1 and ψ_2 are the electrostatic potentials on the particle and gel surfaces, respectively. The value of ψ_1 was assume to be the same as the ζ potential determined by electrophoresis (-50 mV). ψ_2 was estimated based on Donnan equilibrium between the gel and outer phase ($\psi_2 = \psi_s$ Fig.S1), given by

$$y_s + (\alpha - 1)\frac{2}{Q}\cosh y_s = ln\left[\sqrt{\left(\frac{Q}{2}\right)^2 + 1} + \frac{Q}{2}\right] - \frac{\sqrt{\left(\frac{Q}{2}\right)^2 + 1} - \alpha}{\frac{Q}{2}}$$

Here $y_s = -(e_0\psi_s)/(k_BT)$, $Q = q/n_i$, and $\alpha = (\varepsilon_o n_0)/(\varepsilon_i n_i)$ [e_0, the elementary charge; q, number density of the fixed charge in gel; n_i and n_o, ion concentrations inside and outside the gel; ε_i and ε_o, permittivity of the gel and of medium, respectively]. q value was calculated from dissociation constant of AAc monomer. The total interaction potential $U = U_e + U_{vdW}$ calculated at various values of [NaCl] (5–50 mM) is shown in Fig.S2. The potential curves had secondary minima, which became deeper on increasing [NaCl]s. The depth of the potential is 1 k_BT at $x \sim 10$ nm, at [NaCl] = 50 mM. Figure S3 shows the area fraction ϕ of adsorbed particles plotted against [NaCl]. The ϕ value increased on increasing [NaCl], as expected from the potential curves.

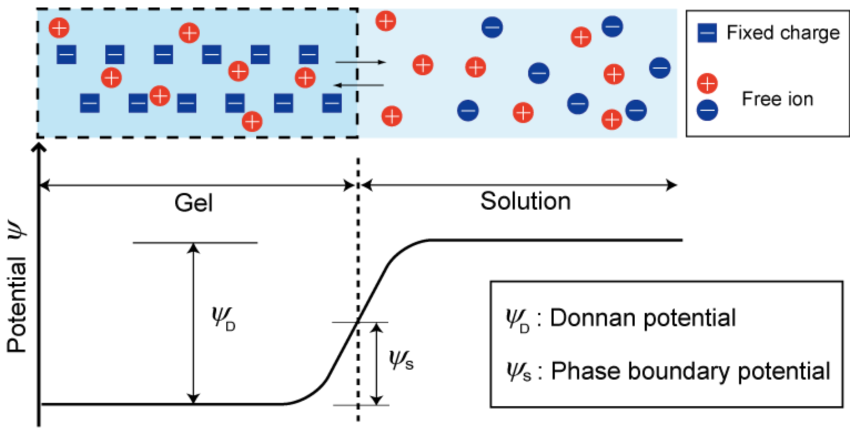

Figure S1 An illustration of Donnan potential and surface potential of charged gel.

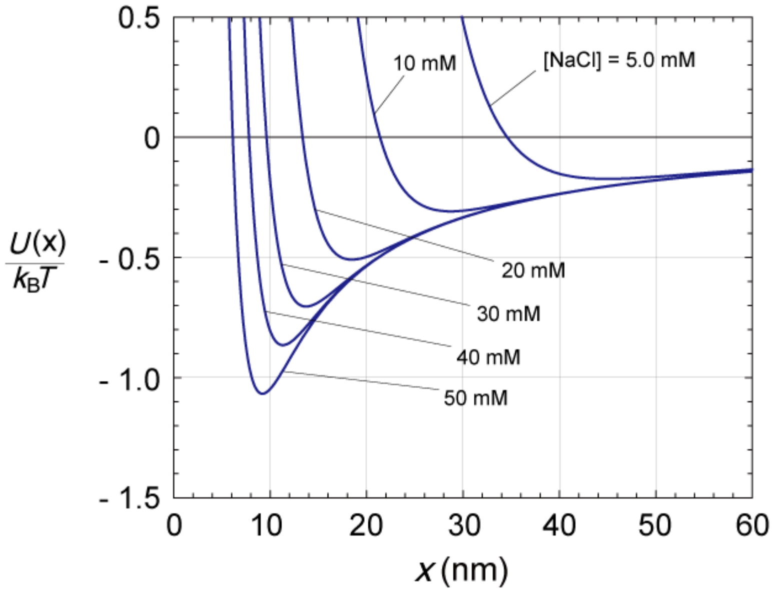

Figure S2 Influence of [NaCl] on the interaction potential $U(x)$ between the charged particle and like charged gel.

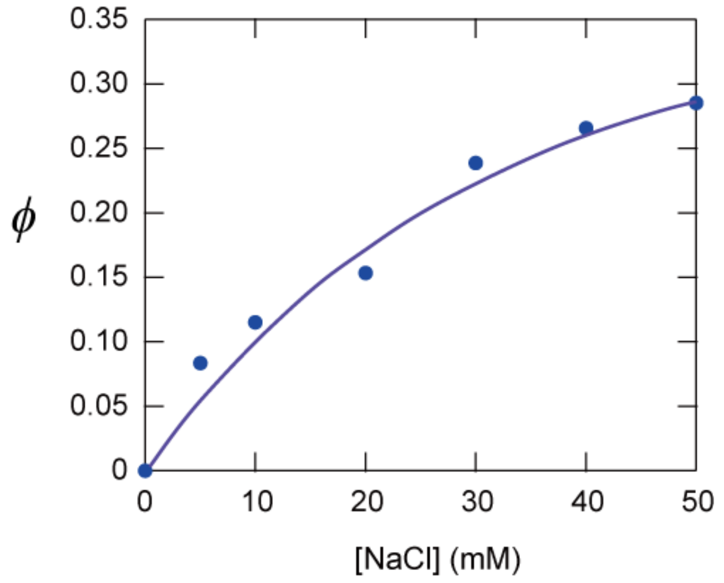

Figure S3 ϕ *vs*. [NaCl] plot for the P(AAm-AAc) gel.

B. Equilibrium sizes of PAAm-based gel and PDMA gel at various values of C_{EG}.

Figure S4 shows diameters of disk shaped PAAm-based gel [taken from reference 29] and PDMA gel in equilibrium swelling at various values of C_{EG} (reduced by the diameter in pure water, d_w). The sample size did not significantly vary on changing C_{EG} for both gel samples.

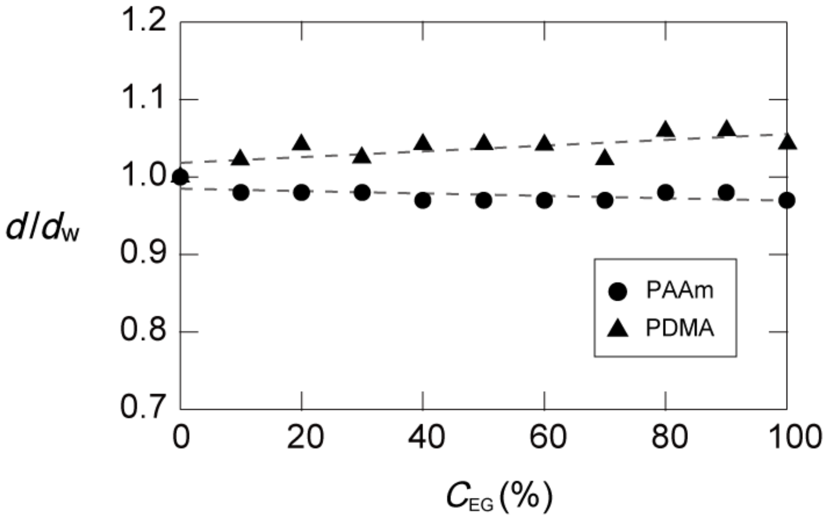

Figure S4 Equilibrium swelling ratios for two kinds of gels at various values of C_{EG}.

C. Estimation of some parameters in adhesion contact theories

Figure S5 shows the Maugis parameter λ vs C_{EG} plot for three values of δ (sample: PAAm gel, E_2 = 6.3kPa). In Figure S6, a_0 and d_0 calculated based on JKR, DMT, and MD theories are presented. Figure S7 presents U_{de} at three values of δ and U_{el}, U_a, and $U_{de} = U_{el} + U_a$ at δ = 2nm, plotted against C_{EG}.

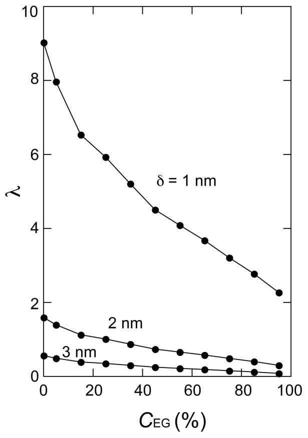

Figure S5 λ vs C_{EG} plot for three values of δ.

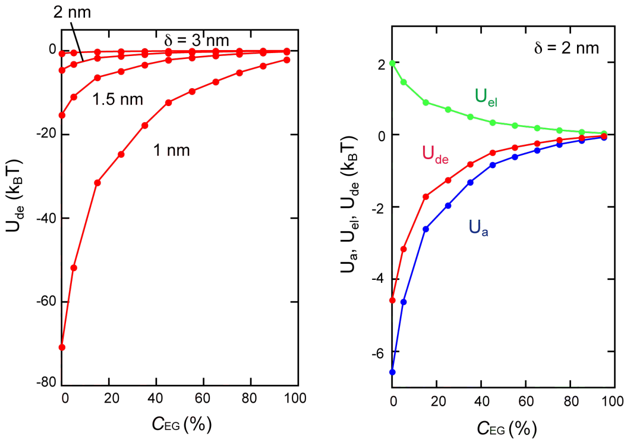

Figure S7 (left) $U_{de} = U_{el} + U_a$ at three values of δ. (right) U_{de}, U_{el} , and U_a plotted against C_{EG}.

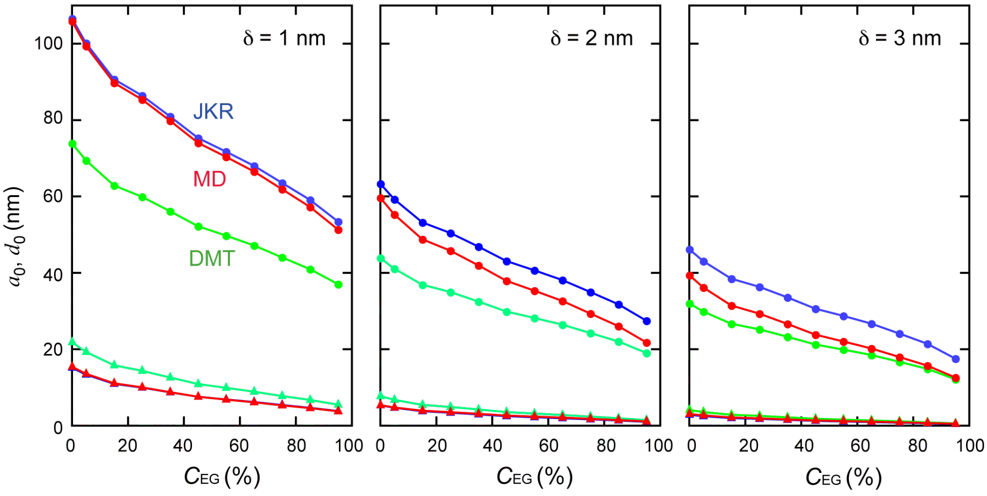

Figure S6 a_0 (circles) and d_0 (triangles) calculated based on JKR, MD, and DMT theories plotted against C_{EG} for three values of δ.
